# Unraveling the human salivary microbiome diversity in Indian populations

**DOI:** 10.1371/journal.pone.0184515

**Published:** 2017-09-08

**Authors:** Anujit Sarkar, Mark Stoneking, Madhusudan R. Nandineni

**Affiliations:** 1 Laboratory of Genomics and Profiling Applications, Centre for DNA Fingerprinting and Diagnostics (CDFD), Hyderabad, Telangana. India; 2 Graduate studies, Manipal University, Manipal. Karnataka, India; 3 Department of Evolutionary Genetics, Max Planck Institute for Evolutionary Anthropology, Leipzig, Germany; 4 Laboratory of DNA Fingerprinting Services, Centre for DNA Fingerprinting and Diagnostics (CDFD), Hyderabad, Telangana. India; MJP Rohilkhand University, INDIA

## Abstract

The importance of studying the salivary microbiome has been highlighted for its connection to health and disease and as a potential tool for supplementing human genetic diversity studies. While the salivary microbiome has been studied in various world populations, Indian populations have not been examined. We therefore analyzed microbiome diversity in saliva samples from 92 volunteers from eight different sampling locations in India by amplifying and sequencing variable regions (V1 and V2) of the bacterial 16S rRNA gene. The results showed immense bacterial richness in Indian populations; we identified 165 bacterial genera and 785 unique Operational Taxonomic Units (OTUs), with substantial sharing among the populations. There were small, but significant correlations in the abundance of bacterial genera in sampling locations from the same geographic region. Most of the core OTUs detected were also observed previously in other populations, but Solobacterium spp., Lachnoanaerobaculum spp. and Alloprevotella spp. were observed to be a component of the saliva microbiome unique to Indian populations. Importantly, nine bacterial genera were observed that were not listed in the Human Oral Microbiome Database (HOMD). These results highlight the importance of analyzing underrepresented populations like those of India.

## Introduction

The human body harbors a large number of microbial cells, organized in complex communities termed microbiota, which was previously thought to exceed the human cells by ten times [1]. A recent study has revised this estimate and proposed that the number of bacterial and human cells in the human body is approximately similar [2]. The bacterial communities differ in their density across various sites in the human body as well as in taxonomic composition and stability [3]. The microbiota of a person differs more at different sites across the body as compared to the interpersonal variation of the microbiome across similar sites [3]. More than 1000 bacterial species, including both disease-causing and health-promoting bacteria have been discovered in the human body, and recent studies have suggested an association of the microbiome imbalance with the health status and predisposition to certain diseases in humans [4,5]. Thus, it is important to understand the bacterial community constituting the “normal” human microbiome in healthy humans [6–9] and its variation across populations. Further, microbiome diversity has been shown to provide insights into the population structure and migratory patterns of humans, highlighted particularly by the studies on *Helicobacter pylori*, a microbe associated with the human gut; the genetic diversity of *H*. *pylori* mirrors human phylogeography and historical migrations [10,11].

The importance of the oral microbiome in the general health status of an individual, and specific bacterial species associated with oral disease have been established [12,13]. A major source for studying oral microbiome is saliva, whose ease of collection and non-invasiveness of the procedure make it an excellent system to study the oral microbiome [14]. Though different world populations have been studied to assess the diversity in the composition of the saliva microbiome [15–17], the same for the diverse populations of India is largely unknown, albeit recently, studies characterizing the gut microbiome [18–20] and salivary microbiome [21] in Indian populations have been reported.

In the present study, we have analyzed the salivary microbiome variation by employing a massively parallel sequencing approach to study the variable regions (V1 and V2) of the bacterial 16S ribosomal RNA (rRNA) gene from 92 healthy volunteers originating from eight different locations representing three geographic regions in India. We explored the diversity within and between each population and present a detailed analysis of the variation in the salivary microbiome among the geographical regions of India. The results demonstrate that the Indian populations display a high bacterial richness along with substantial sharing of the salivary microbiome among different populations.

## Materials and methods

### Sample collection and DNA extraction

All of the participating volunteers provided written informed consent and the study was approved by the Institutional Bioethics Committee of the Centre for DNA Fingerprinting and Diagnostics (CDFD). All the methods were carried out in accordance with the approved guidelines. Saliva samples (up to 2 mL) from unrelated healthy adult volunteers were collected in an unstimulated fashion by requesting them to spit in tubes containing 2 mL of lysis buffer [22]. The tubes were sealed and immediately transported to the laboratory at room temperature for DNA extraction. The region-wise details of the sample numbers are summarized in Table 1 and the corresponding sampling locations are depicted in S1 Fig. The detailed oral health status of each volunteer was not clinically investigated, however, none of them was suffering from obvious oral lesions and none reported that they were under any antibiotic treatment. DNA was extracted according to the protocol published previously [22].

**Table 1:** Distribution of reads, bacterial genera and OTUs across various geographical locations (States) in India.

Geographic region (States)	Code	Region	Number of samples	Total sequence reads	Average reads per sample	Total unique genera	Number of Bacterial genera observed per sample	Number of OTUs observed (non-unique)
Jammu and Kashmir	JK	North	12	285116	23759	85	35–57	224804
Uttarakhand	UT	North	12	407044	33920	99	37–64	319889
Jharkhand	JH	East	11	368243	33476	96	41–63	281027
West Bengal	WB	East	14	368035	26288	104	38–58	280897
Assam	AS	East	10	299481	29948	92	37–68	240921
Andhra Pradesh	AP	South	10	267224	26722	93	40–62	203843
Telangana	TS	South	12	427990	35665	80	38–56	336072
Tamil Nadu	TN	South	11	343522	31229	79	39–56	276042

### PCR amplification of the bacterial 16S rRNA gene

The highly informative variable regions (V1 and V2) were amplified using the primers reported previously [23]. The PCR was carried out in 50 μL reaction volume, including 32.5 μL of ddH_2_O, 10 μL of 5X HF buffer, 1 μL of 10 mM dNTPs, 0.5 μL of 50 mM MgCl_2_, 2 μL each of 10μM forward and reverse primers and 1 unit of Phusion DNA polymerase (Thermo Scientific, USA) and 40 ng of DNA at the following PCR conditions: initial denaturation at 98°C for 30 seconds followed by 30 cycles of 98°C for 15 seconds, 66°C for 25 seconds and 72°C for 30 seconds and final extension at 72°C for 10 minutes.

### Sequencing on Illumina MiSeq platform

The PCR products from the variable regions were processed for parallel-tagged sequencing on the MiSeq platform (Illumina, CA, USA) following the procedures described previously [24]. Sample-specific barcode sequences were ligated at both ends of the PCR products, quantified using NanoDrop 2000c Spectrophotometer (Thermo Fisher Scientific, USA) and equimolar quantities of each amplified product was pooled. The library pool was then quantified using the Agilent 2100 Bioanalyzer (Agilent Technologies, Inc. USA), followed by amplification and paired-end sequencing on the MiSeq platform (2 x 250 bp) according to the manufacturer’s instructions.

### Sequence analysis

The reads obtained were filtered to discard sequences with an average Phred score < 30 and sequences containing incorrect barcodes and/or lacking primer sequences. Overlapping paired-end reads were merged to reconstruct the full-length sequences of the target regions. The reads containing ambiguous bases (N), homopolymer stretches (>8 bases) and reads either too small (<330 bases) or large (> 430 bases) were discarded using the software mothur [25]. The filtered reads were imported to USEARCH [26] and were dereplicated, followed by subsequent removal of singletons and identified chimeras. The processed reads were then clustered at 97% homology to identify the species-level Operational Taxonomic Units (OTUs) and the reads were mapped to the filtered OTUs to determine the exact count of each OTU in each sample.

To identify the bacterial genera present in each sample, the filtered reads obtained from mothur were aligned, followed by BLAST comparison at 80% cutoff (minimum bootstrap value to assign a read to a bacterial genus confidently) against the 16S Ribosomal Database Project (RDP-II) [27] using the Wang method implemented in mothur [28]. For the purpose of Unifrac analysis, the identified OTUs were aligned in mothur using the 16S rRNA Silva database as template followed by constructing a phylogenetic tree using the generalized time-reversible (GTR) model available in Fasttree [29] and processed in Figtree ver.1.3.1 (http://tree.bio.ed.ac.uk/software/figtree/) for visualization, which was subsequently used in GUniFrac [30]. Calculation of alpha and beta diversity indices and rarefaction analyses were carried out by the ‘vegan’ package while partial correlation analyses was carried out by the ‘GeneNet’ package, both implemented in R [31,32]. To calculate the diversity indices for each sampling location, 10000 reads from 10 random samples from each population were utilized. For calculating indices across three geographic regions, we took; nine random samples from Jammu & Kashmir and Uttarakhand to represent North India; six samples each from Jharkhand, West Bengal and Assam to represent East India, and six samples each from Andhra Pradesh, Telangana and Tamil Nadu to represent South India. The Venn diagram was plotted with the help of the ‘VennDiagram’ package in R [33] and geospatial analysis involving the spatial distribution of the salivary microbiome across various populations was carried out with the Adonis function in GUniFrac. For the geospatial analysis, geographical location, annual mean temperature, latitude, population density and altitude were considered as the experimental variables. The details of the experimental variables at each sampling location are mentioned in S1 Table.

To identify the bacterial genera not reported in the Human Oral Microbiome Database (HOMD), a threshold of 90% sequence identity was set to correctly identify the genera. Briefly, all of the 785 OTUs were tested against the HOMD and the 16S RDP II databases. The sequences which could not be assigned to a genus in HOMD (at 90% identity) but were clearly identified in the RDP II project (≥ 90% sequence similarity) were considered as the probable candidates which could be unique or novel in the oral cavity of the Indian populations. For comparing the salivary microbiome diversity with a previous study of Alaskans, Africans, and Germans [17], 10 samples were randomly selected from the 92 samples such that at least one sample from each of the eight sampling locations was included. From the previous study as well, 10 random samples were selected representing the Alaskans and Africans; the samples were selected in such a way so as to ensure that all the sub-populations are similarly represented. As the German population had only 10 individuals, all of them were included for rarefaction analysis. Here also, equal numbers of reads were sampled from each individual to account for differences in the sequencing depth across the samples in the previous and the current studies. For all statistical calculations involving multiple comparisons, Sidak correction [34] was applied.

## Results

### Sequence processing

A total of 2,766,655 reads from 92 samples were obtained, and their corresponding distribution across the studied geographical regions is shown in Table 1. The USEARCH pipeline for OTU clustering generated 785 unique OTUs to which 84.6% of the filtered reads were mapped and clustered successfully. Upon classification of all the reads, a total of 165 bacterial genera was observed.

### Microbiome diversity at the genus level

A total of 9.4% of the filtered sequences could not be classified up to the genus level and 0.89% of the sequences did not match any of the sequences in the RDP II database. The number of processed reads and the corresponding counts of bacterial genera and OTUs detected in each of the populations are shown in Table 1, and the distribution of major bacterial genera represented as pie charts across the sampling locations is shown in S1 Fig. The sampling sites were further grouped according to their geographical locations within India into three regions viz., (Table 1) Northern India (states of Jammu & Kashmir and Uttarakhand), Eastern India (states of Jharkhand, West Bengal and Assam) and Southern India (states of Andhra Pradesh, Telangana and Tamil Nadu). The West Bengal (East India) population displayed the highest number of unique genera, while the Tamil Nadu (South India) showed the least (Table 1). Rarefaction analysis (S2 Fig) for all the populations showed that the sequencing approach and depth was sufficient to ascertain the bacterial richness.

**Fig 1:**
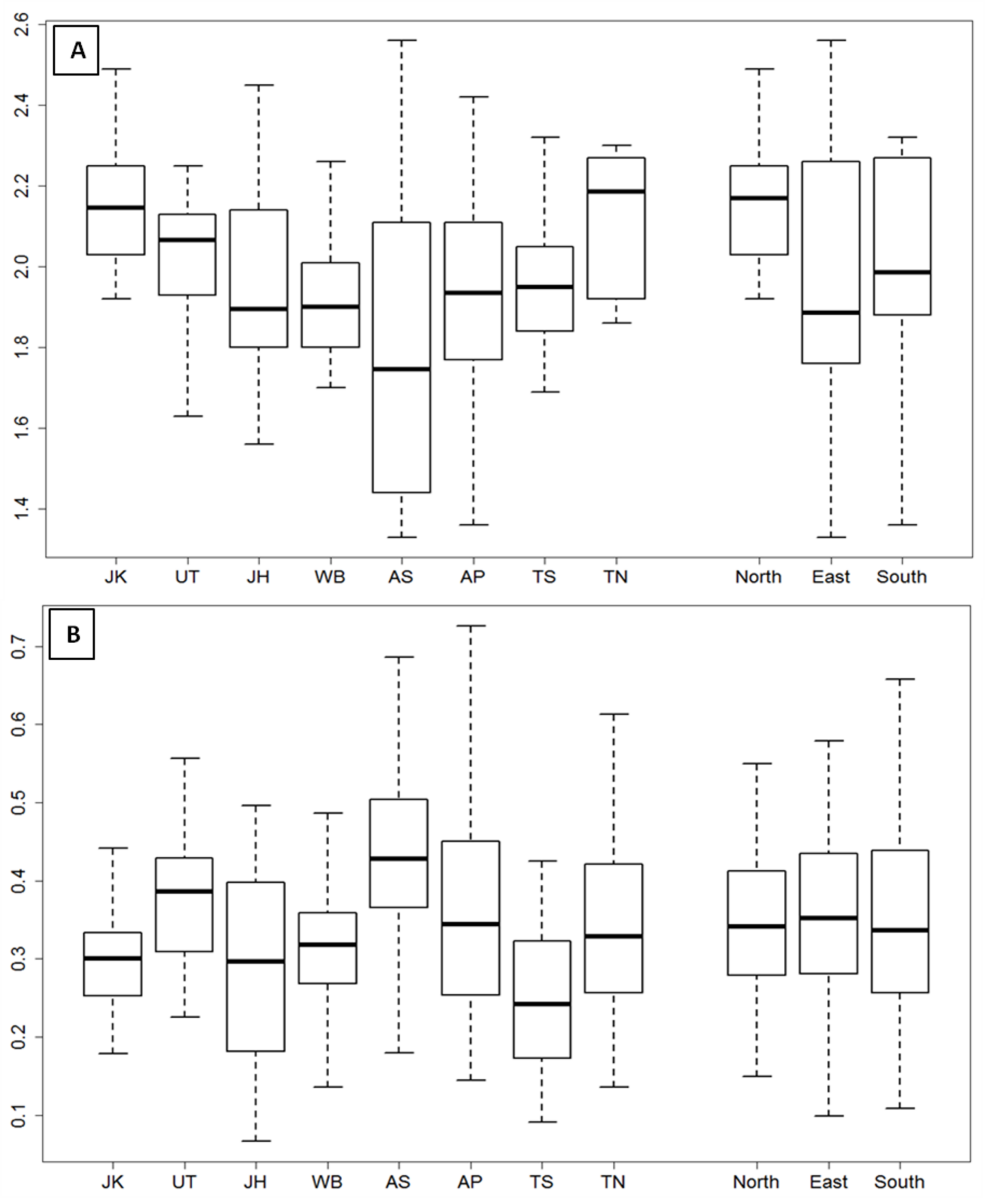
Box plot comparison of alpha and beta diversity analysis across populations and geographic regions at the genera level. X-axis denotes the population studied while Y-axis denotes the corresponding Shannon-Weaver index (A) and Sorensen index (B) representing the alpha diversity and beta diversity, respectively. Samples belonging to the same biogeographic region (as listed in Table 1) have been merged together to estimate the diversity among regions.

Diversity indices to apportion the observed variability within and between populations were obtained by calculating the Shannon-Weaver index [35], corresponding to alpha diversity, and the Sorensen index [36], corresponding to the beta diversity, as shown in Fig 1. The populations from North India displayed the highest alpha diversity (Shannon index) followed by South and East Indian populations, whereas the Sorensen index was highest for the Eastern India populations and approximately similar for populations from North and South India.

Mann-Whitney U tests were performed to examine if the alpha and beta diversities differed significantly within and among biogeographic regions; no significant difference in the alpha diversity was observed within or between geographic regions. When the corresponding beta diversity values were compared, populations from North India showed significant differences among themselves (p = 0.0001). Among the East Indian populations, Assam showed high inter-individual diversity and therefore, showed differences with both Jharkhand (p = 2.35 x 10^−6^) and West Bengal (p = 1.61 x 10^−6^), whereas Jharkhand and West Bengal were not significantly different from each other (p = 0.37). Within the South Indian populations, the samples from the state of Telangana possessed low inter-individual diversity as compared to others, and showed significant differences with both Andhra Pradesh and Tamil Nadu samples (p = 4.99 x 10^−5^ and p = 0.0004, respectively). However, there were no significant differences between regions. The results indicate that, overall, although the ‘within-individual’ diversity (alpha diversity) did not differ much within any population or region, the ‘between individual-diversity’ (beta diversity) was substantially higher in samples from Uttarakhand, Assam and Andhra Pradesh, and lowest in the Telangana samples. Overall, the beta diversity was lowest in populations from East India, while it was relatively similar in populations from North and South India.

### Microbiome diversity based on the abundance of bacterial genera

*Streptococcus* spp. was observed to be the major bacterial genus, with approximately 35% contribution to the North Indian populations, 42% of the East Indian populations and 38% of the South Indian populations. A heat map of the major bacterial genera (abundance >1% in at least one sample) is shown in S3 Fig. The genera of *Prevotella*, *Fusobacterium*, *Veillonella*, *Leptotrichia* and *Granulicatella* were identified as other major contributors of the salivary microbiome in all the populations. Interestingly, *Chromobacterium* spp. was found to be highly abundant (~14%) in a few samples from the Assamese population (East India); otherwise the frequency reached about 1.5% in Uttarakhand and less than 0.04% in all other populations.

Upon comparing the proportion of the common bacterial genera (greater than 0.05% in at least one population), several bacterial genera (including *Gemella*, *Moryella*, *Stenotrophomonas* and *Streptobacillus)* were found to occur in varying abundance in different populations (S2 Table). Finally, we observed enrichment of a few bacterial genera in some of the populations, including: *Atopobium*, *Megasphaera* and *Prevotella* in the Tamil Nadu samples; *Streptobacillus* and *Bacillus* in the Telangana samples; and *Stenotrophomonas* in the Uttarakhand and Assam samples. The distribution of the abundant genera at the regional level also identified interesting candidates showing statistically significant differences in abundance among the studied regions (S3 Table). In particular, *Megasphaera* was found to be considerably lower in frequency in the East Indian populations, whereas *Stenotrophomonas* was significantly lower and *Neisseria* and *Streptobacillus* significantly higher in samples from South India.

### Correlation among the samples based on shared bacterial genera

To measure the degree of overlap in the salivary microbiome in these samples, the corresponding correlation coefficients among all pairs of samples (correcting for multiple comparisons) were calculated. Within each sampling location, significant correlations were observed at all places except in Assam (r = 0.69, p = 0.003, p_adj_ cutoff = 0.001). When the populations within each geographic region were compared, samples from Jammu & Kashmir and Uttarakhand in North India were correlated (r = 0.8, p = 8.14 x 10^−10^). In the East region, Jharkhand and West Bengal showed significant correlation (r = 0.89, p = 3.6 x 10^−18^), while the samples from Assam were neither correlated to Jharkhand (r = 0.77, p = 0.013, p_adj_ cutoff = 4.66 x 10^−4^) nor with West Bengal (r = 0.77, p = 0.01, p_adj_ cutoff = 3.59 x 10^−4^). In South India, Andhra Pradesh and Telangana samples were significantly correlated (r = 0.84, p = 2.94 x 10^−5^). However, samples from Tamil Nadu were not correlated to either Andhra Pradesh (r = 0.75, p = 0.003, p_adj_ cutoff = 4.6 x 10^−4^), or Telangana (r = 0.74, p = 5.33 x 10^−3^, p_adj_ cutoff = 3.88 x 10^−4^). Finally, upon comparing samples between regions, only a subtle Pradesh (r = 0.75, p = 0.003, p_adj_ cutoff = 4.6 x 10^−4^), or Telangana (r = 0.74, p = 5.33 x 10^−3^, p_adj_ cutoff = 3.88 x 10^−4^). Finally, upon comparing samples between regions, only a subtle correlation among North and South India was observed (r = 0.79, p = 2.5 x 10^−5,^ p_adj_ cutoff = 6.6 x 10^−5^) Overall, the samples showed slightly higher correlations within the regions in comparison to the correlation across samples belonging to different geographical regions.

### Interactions between bacterial genera

Partial correlation analysis was carried out to address the extent of interactions between bacterial genera based on their relative abundance [17]. The existence of bacterial interaction networks was explored by constructing and comparing the partial correlation networks among the samples using a false discovery rate (FDR) of 0.01 in each geographical region. A total of 226, 872 and 332 significant interactions were observed in the populations from North, East and South India, respectively. For bacterial genera with a frequency >0.05% in a region, the number of interactions in North, East and South India were one, nine and one, respectively. None of the interactions was observed to be common across all regions for bacterial genera with greater than 0.05% abundance (S4 Fig).

### Phylogenetic analyses

**Fig 2:**
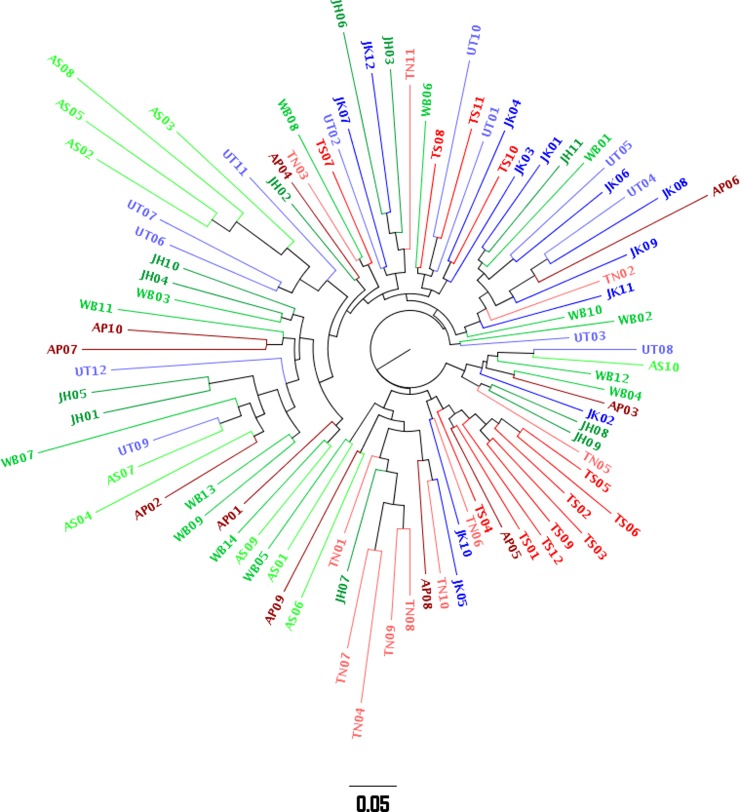
Phylogenetic tree based on the Unifrac distance. Each tip denotes a sample while the colour of the tip and its corresponding branch indicate the affiliated population. Sampling locations belonging to each geographic region (North, East and South) have been assigned different shades of the same colour.

The Unifrac metric was utilized to measure the pairwise distance between each pair of samples, based on the relative amount of shared sequence phylogeny, and the corresponding 2D non-metric multidimensional scaling (NMDS) plot was constructed (S5 Fig). A phylogenetic tree based on the unweighted Unifrac distances was also constructed and is shown in Fig 2. It was observed that even while the samples from the same population/region tend to cluster together to some extent in these analyses, considerable overlap was also observed among samples from different populations or regions. The results were in agreement with the diversity and correlation analyses.

### Comparison with HOMD

By considering a sequence identity threshold of 90%, we examined the existence of new bacterial genera in our study which were not previously reported in the HOMD. Upon classifying the 785 OTUs with the HOMD, a total of 54 OTUs were observed with sequence identity of less than 90%. Also, the abundance of these 54 OTUs were observed to be significantly lower (0.042%) than for those listed in HOMD (0.13%, p = 2.36 x 10^−9^) suggesting that sequencing depth plays a role in identifying the rarer sequences in the current study. The 54 OTUs could be grouped into 24 genera (assuming a threshold of 90% sequence identity for genera assignment); nine of these were not reported in the HOMD viz., *Meiothermus* spp., *Hydrocarboniphaga* spp., *Streptobacillus* spp., *Brevibacterium* spp., *Brevibacillus* spp., *Weissella* spp., *Aeromonas* spp., *Chromobacterium* spp. and *Aeribacillus* spp.

### Core microbiome

In order to test for the existence of a putative core microbiome in the Indian population, the distribution of all of the 785 OTUs was examined across the samples. An extensive sharing of OTUs was observed across all the regions, as shown in S6 Fig. The populations from North India shared 683 and 675 OTUs with the East and South Indian populations, respectively, while the East India populations shared 703 OTUs with the South Indian populations. A total of 660 OTUs was found to be shared in all three geographic regions, of which 37 OTUs were found in all the individuals and hence could comprise a putative core microbiome for Indian populations. In order to further understand the structure of this putative core microbiome, the corresponding taxonomy of all 37 OTUs were determined and compared with previous studies [17,37,38]. All the 37 OTUs could be assigned to 12 bacterial genera. A summary of these results is shown in S4 Table. Among the bacterial genera, 19 out of 165 were observed in all the samples. However, all of them were among the more abundant genera and were previously reported in HOMD.

### Geospatial analysis

Geospatial analysis was carried out to investigate the association of the salivary microbiome composition at the genus level with common physical factors, including geographical location, latitude, altitude, mean annual temperature and population density. Permutational multivariate analysis of variance (PERMANOVA) was performed using the unweighted unifrac distance between samples. Geographical location was observed to be a major contributor to the variance (~ 19%, p = 0.001), followed by annual mean temperature and latitude, which explained approximately 3.2% (p = 0.004) and 2.4% (0.02) of the variance, respectively. Population density and altitude had the least effect, explaining only 1.9 (p = 0.07) and 1.3% (p = 0.21) of the salivary microbiome variation respectively, and were not statistically significant. A corresponding plot is shown in S7 Fig.

To further explore the differential occurrence of bacterial genera in the salivary microbiome of Indian populations, we carried out an Analysis of similarity (ANOSIM) based upon the frequency of bacterial genera among the samples. We observed approximately 15.5% difference across the geographic locations (p = 0.001). Based on both genera distribution and unifrac metrics on OTU abundances, small but significant differences in the microbiome composition were observed across the geographical locations.

### Comparison with previous studies

We compared the bacterial genera richness observed in this study to that reported previously [17] from three worldwide populations from Alaska, Germany, and Africa. Subsampling an equal number of reads from the Alaskan, German, African, and Indian data followed by rarefaction analyses (S8 Fig) showed that fewer bacterial genera were observed in the Indian populations than in the others: 39 genera in the Indian population, compared to 39, 43 and 46 genera in Alaska, Germany and Africa respectively.

## Discussion

This study describes the salivary microbiome composition in 92 samples from eight different locations comprising three major geographic regions in India. Employing a high throughput sequencing approach, the informative regions (V1 and V2) of the phylogenetic marker 16S rRNA gene were used to analyze the salivary microbiome diversity in these populations. The findings support the previous observation that the salivary microbiome is among the most diverse in the human body and possesses substantial richness [7]. We observed 165 unique bacterial genera and 785 different OTUs, which was higher than those found in other world populations, including Africans [17,39], where up to 127 bacterial genera were observed. However, rarefaction analyses by subsampling an equal number of reads from the Indian and other world populations [17] suggests that the bacterial richness of Indian populations is in fact similar to that in Africans, Alaskans and Germans (S8 Fig). As the rarefaction analyses were carried out with a much smaller subset of reads for the Indian population, only the more abundant genera were detected. This indicates the importance of deep sequencing to detect the rarer bacterial genera which were previously undetected [17]. For comparison with the previous study [17], only a subset of samples and reads have been utilized to minimize the bias arising from different sample size and sequencing depth while comparing the two datasets. It can be noted that since the subsets of the datasets were constructed so as to incorporate representatives from all the subpopulations, they might not be truly random. However, to accommodate the bacterial richness arising from different subpopulations in each dataset, at least one sample from each subpopulation was considered. Similarly, for calculating diversity indices, we have created subsets of our dataset both for the number of samples and number of reads to eliminate the chances of incorrect inferences drawn due to the variable number of samples in each sampling location and region and also to control for the effects of differential sequencing depth in each sample. This way, although some information might be lost due to non-utilization of all the available data, an unbiased estimate of the diversity indices has been calculated.

The ability to pick up even extremely rare microbial species by the deep sequencing approach adopted in this study can be gauged by the fact that 54 OTUs observed in this study were not previously reported in the Human Oral Microbiome Database (HOMD). We further show that these OTUs were in fact significantly less abundant than other genera, further indicating the contribution of sequencing depth in detecting the novel bacterial genera in the current study. We also observed nine novel bacterial genera previously unreported in HOMD, highlighting the importance of studying underrepresented populations for a better understanding of the human salivary microbiome. Even though the rarefaction analyses indicated lesser bacterial richness in the Indian populations as compared to Africans, and Germans, the sequence processing steps might have affected the results as more stringent parameters were utilized here. For example, reads containing ambiguous bases and homonucleotide repeats larger than eight were discarded here, but were retained in the previous study [17].

We compared our results with a recent study [17] to further explore the effects of sequencing depth for two reasons. First, the previous study encompasses an entirely different and diverse set of human populations from Alaska, Germany and Africa. Second, the same variable region (V1 and V2) of the 16S rRNA gene was examined in both these studies, thereby nullifying the effects of differential microbiome detection based on target region [40]. Interestingly, although the detection of the Enterobacteriaceae family (*Kleibsiella* spp., *Enterobacter* spp., etc.,) was previously found to be specific to the African populations, they were also detected in the Indian samples, albeit in lower abundance as compared to the Africans. This observation further emphasizes the role of sequencing depth in discovering the rarer bacteria, as their ready detection in African populations might be explained by the favorable climatic and physiological conditions, resulting in higher abundance as discussed previously [17].

Similar to a previous study [16], latitude, a function of distance from the equator, was observed to explain a significant (albeit only a small) fraction of the variance in the salivary microbiome. We observed annual temperature to be a major contributor towards the composition of the salivary microbiome. Despite the temperature of the saliva being relatively stable, its exposure to the external environment might affect its temperature with seasonal changes. Consequently, the higher prevalence of the Enterobacteriaceae family in African populations was speculated to be associated with the higher external temperature which promotes their growth [17]. Additionally, sudden changes in the outside temperature owing to seasonal variation might also induce corresponding changes in the salivary microbiome.

All the significant correlations observed were largely regional rather than global, as reported previously [17], probably suggesting a greater role for the local environment (including substrate availability and ethnicity) in determining the degree of interaction. The correlation analysis of the populations and regions clearly suggest that they are more correlated within than between populations, even though substantial sharing of the microbiome was observed across the populations. It also indicates that geographical proximity might enhance sharing of the oral microbiome. Diversity indices, unifrac studies and correlation studies among samples indicate higher sharing of the salivary microbiome within vs. between each region. A potential explanation for these results is food habits, which vary between regions. However, the contribution of food habits towards shaping the microbiome has been equivocal, with a few studies supporting the role of food habits as a key contributor to microbiome composition [20,39], while others observed a minimal role [41,42]. Although detailed information about the food habits of the participants in the current study was not recorded, substantial sharing of the bacterial genera suggests a minimal effect of any variation in the diet on the salivary microbiome.

As the samples displayed relatively high homogeneity of microbial genera and did not cluster entirely by geography, the current study is in agreement with a previous study [16], which suggested a minimal correlation between geographic distances among populations and the corresponding salivary microbiome composition. However, small but significant differences were observed among the sampling locations, thereby indicating the role of geography as well, which was supported by unifrac and phylogeographic analysis (Figs 2, S5 and S7). The inclusion of urban samples with similar socio-economic levels might have further reduced the diversity observed across the locations. Overall, the general perception of substantial richness and high sharing of the salivary microbiome [7] is corroborated in the current study, as reflected by the higher alpha diversity (representing high richness) and lower beta diversity (representing less inter-individual variation, and therefore higher sharing). In the previous study involving the gut microbiome in Indian populations [20], the authors observed significant differences across the studied populations primarily because the samples were mostly from isolated tribes with a well-defined culture, life-style and food habits. In contrast, the volunteers studied here have similar socio-economic backgrounds and possible overlap in food habit, which might have reduced the overall microbiome variation. Further, as the saliva microbiome is more exposed to the natural environment than the gut microbiota, the governing factors defining the overall composition of the salivary and the gut microbiome might be different.

Some aspects of our study do warrant caution in the interpretations. For example, although the volunteers were all healthy at the time of saliva collection, it is possible that undetected differences in oral health might be influencing bacterial diversity. However, a recent study found a negligible impact of dental caries on the salivary microbiome [43]. Focusing on only a portion of the 16S rRNA gene (in order to utilize the NGS capacity) instead of analyzing the entire gene could also limit our ability to assign the sequences to different species, as reported previously [20]. Though studies have shown that V3 and V4 regions of the 16S rRNA gene provides greater robustness for microbiome studies, the V1 and V2 regions were observed to provide more phylotypes [23] and therefore, were targeted here. Recently, improved library preparation protocols were suggested for 16S rRNA amplicon studies [44], however, at the time of designing this study, such protocols were not available. As all samples were analyzed with the same library preparation protocol, this is not expected to influence the differences observed between populations. Also, the relatively low sample size per population (N = 10 to 14) might have reduced the discriminatory power of our study.

This study provides a snapshot of the salivary microbiome variation of the various Indian populations at a single time point, which then raises the question of temporal variation in the saliva microbiome. Although the gut microbiome has been found to be relatively stable to seasonal variations [45], the saliva microbiome could be comparatively vulnerable owing to its higher exposure to the environment. However, various studies have shown that the saliva microbiome is stable over time [46–48]. Hence, the differences between the individuals observed in this study are likely to be independent of the time of sampling.

The search for a core microbiome in the Indian populations showed significant overlap of OTUs from the various geographic regions. A total of 640 out of 785 OTUs were shared across all the regions, thereby exhibiting high sharing of OTUs among populations, an observation similar to that based on bacterial genera. The 37 core OTUs observed in all samples in the current study, many of which were also previously described for other populations (S4 Table), supports the existence of a common set of bacterial genera across various worldwide human populations, including India. Additionally, *Solobacterium* spp. and *Alloprevotella* spp. were found in all the samples in our study but were not a part of the core microbiome in previous studies. Although *Solobacterium* spp. and particularly *Solobacterium moorei* has been associated with halitosis [49], a recent study has observed the occurrence of *Solobacterium* in healthy samples [50], suggesting the occurrence of *Solobacterium* spp. as part of the “normal” salivary microbiome. In addition, *Lachnoanaerobaculum* spp., which was recently isolated from human saliva [51], was observed as a part of the core human microbiome in our study and might be playing a role in glucose metabolism, as reported previously [51]. Several bacterial species from the genus *Streptobacillus*, that are known to be associated with disease phenotypes, were also found to be differentially distributed among the populations and regions examined in the present study. However, as the resolution in this study could only be obtained up to the genus level, their association with the oral health status of the participating volunteers could not be established. The degree to which this differential distribution might contribute to different patterns of disease across Indian populations could be an important focus of further studies. Additionally, collecting detailed information regarding the socio-economic status of the volunteers, food habits and the frequency of food uptake along with detailed oral hygiene practices would be helpful to better understand how the saliva microbiome might both influence and be influenced by an individual’s lifestyle. Given the extraordinary genetic diversity of Indian populations, the Indian subcontinent is a natural laboratory for further such investigations.

## Conclusions

This study represents the first comprehensive survey of the salivary microbiome across Indian populations. It also highlights the importance of studying underrepresented populations, as new bacterial genera can be discovered which were not observed in other populations. Supporting the general perception of high sharing of the salivary microbiome among healthy volunteers, we also find small but significant association towards the geographical locations. The study further emphasizes the importance of deep sequencing in discovering rarer bacterial genera. Although most of the core OTUs detected in this study were also observed previously in other populations, *Solobacterium* spp., *Alloprevotella* spp. and *Lachnoanaerobaculum* spp. could be a component of the core saliva microbiome in only the Indian populations. Overall, this study aids in understanding the composition and distribution of the normal saliva microbiome in human populations, which ultimately might aid in determining the role of the saliva microbime in various disease phenotypes.

## Supporting information

S1 Fig Distribution of major bacterial genera across the sampling locations. Populations are identified and abbreviated as listed in Table 1. Pie charts display the relative abundances of the major bacterial genera across the eight sampling locations and three geographic regions viz., North India (JK and UT represented with blue), East India (JH, WB and AS represented with green) and South India (AP, TS and TN represented with red).
(TIF)

S2 Fig Rarefaction plot to assess the bacterial genera richness as a function of sampled sequences grouped according to sampling locations. The analysis was carried out based on the abundance of various bacterial genera identified by sequencing the 16S rRNA gene (V1-V2 region) in each individual. The X-axis shows the number of randomly sampled sequences from each individual (one curve per individual) while Y-axis represents the mean bacterial richness based upon the bacterial genera identified. The sample code at the right bottom of each plot indicates the population.
(TIF)

S3 Fig Heatmap showing the relative abundance of major bacterial genera across the populations and the corresponding relationships among the samples. Bacterial genera with at least 1% abundance in a sample are represented in each column while individuals (N = 92) are clustered according to their relative distribution of bacterial genera. Colour key indicates the proportion of reads assigned to a genus for each sample. Sample codes; 1–12 (JK), 13–24 (UT), 25–35 (JH), 36–49 (WB), 50–59 (AS), 70–81 (TS) and 82–92 (TN).
(TIF)

S4 Fig Bacterial genera (> 0.05% in at least one region) interaction network across the three regions. Straight line indicates positive interaction and curved line indicates negative interaction. Line colour denotes the corresponding regions: North (blue), East (green) and South (red).
(TIF)

S5 Fig Principal coordinate analysis (PCoA) based on unweighed unifrac distances. The first two components (PC1 and PC2) are shown here (Stress percentage = 17.5). Each sample is represented by a filled circle colored according to its geographical location. Populations from the same geographic region are represented by different shades of the same colour.
(TIF)

S6 Fig A core salivary microbiome, as identified by OTU sharing among the biogeographic regions, represented as a Venn diagram. The distribution of 785 unique OTUs (obtained at 97% clustering) across North (blue), East (green) and South (red) India are shown.
(TIF)

S7 Fig Percentage of salivary microbiome variance explained on average by different factors, using 10000 permutations in a PERMANOVA analysis. (*) shows the association was statistically significant (p<0.05).
(TIF)

S8 Fig Rarefaction analysis to compare the microbial richness between the Africans, Germans, Alaskans [17] and Indians. The analysis was carried out based on the abundance of various bacterial genera identified by sequencing of the partial 16S rRNA gene (V1-V2) in each individual. The X-axis shows the number of randomly sampled sequences from each population while Y-axis represents the mean bacterial richness based upon the bacterial genera identified.
(TIF)

S1 Table Details of the experimental variables at each sampling location used for geospatial analyses of salivary microbiome variation in India. (DOCX)

S2 Table Distribution of bacterial genera (> 0.05% in at least one location) and their corresponding tests of significance (p < 0.001, Sidak correction) for differential prevalence (genera displaying significantly different prevalence are marked in bold). (DOCX)

S3 Table Distribution of bacterial genera (> 0.05% in at least one region) among the geographic regions and their corresponding tests of significance (p < 0.001, Sidak correction) (genera displaying significantly different prevalence are marked in bold). (DOCX)

S4 Table Comparison of the 37 OTUs obtained in all the samples with the core microbiome described in previous studies. (DOCX)
