# Supplementary figures and images for: Unraveling the human salivary microbiome diversity in Indian populations
Source: PLoS One. 2017 Sep 8;12(9):e0184515. doi: 10.1371/journal.pone.0184515 (PMC5590957; doi:10.1371/journal.pone.0184515)

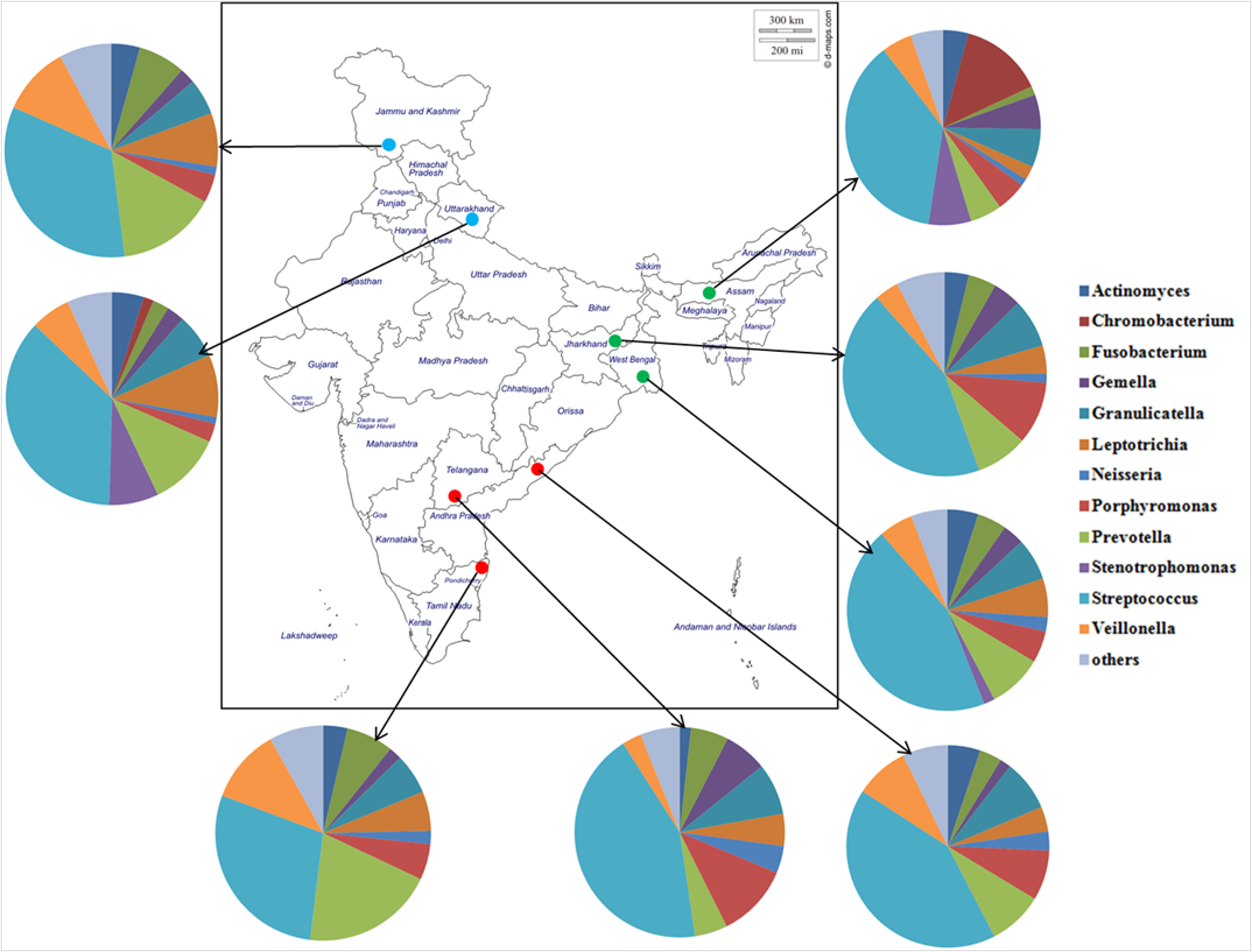

Supplement: S1 Fig — Populations are identified and abbreviated as listed in Table 1. Pie charts display the relative abundances of the major bacterial genera across the eight sampling locations and three geographic regions viz., North India (JK and UT represented with blue), East India (JH, WB and AS represented with green) and South India (AP, TS and TN represented with red). (TIF) [file pone.0184515.s001.tif]

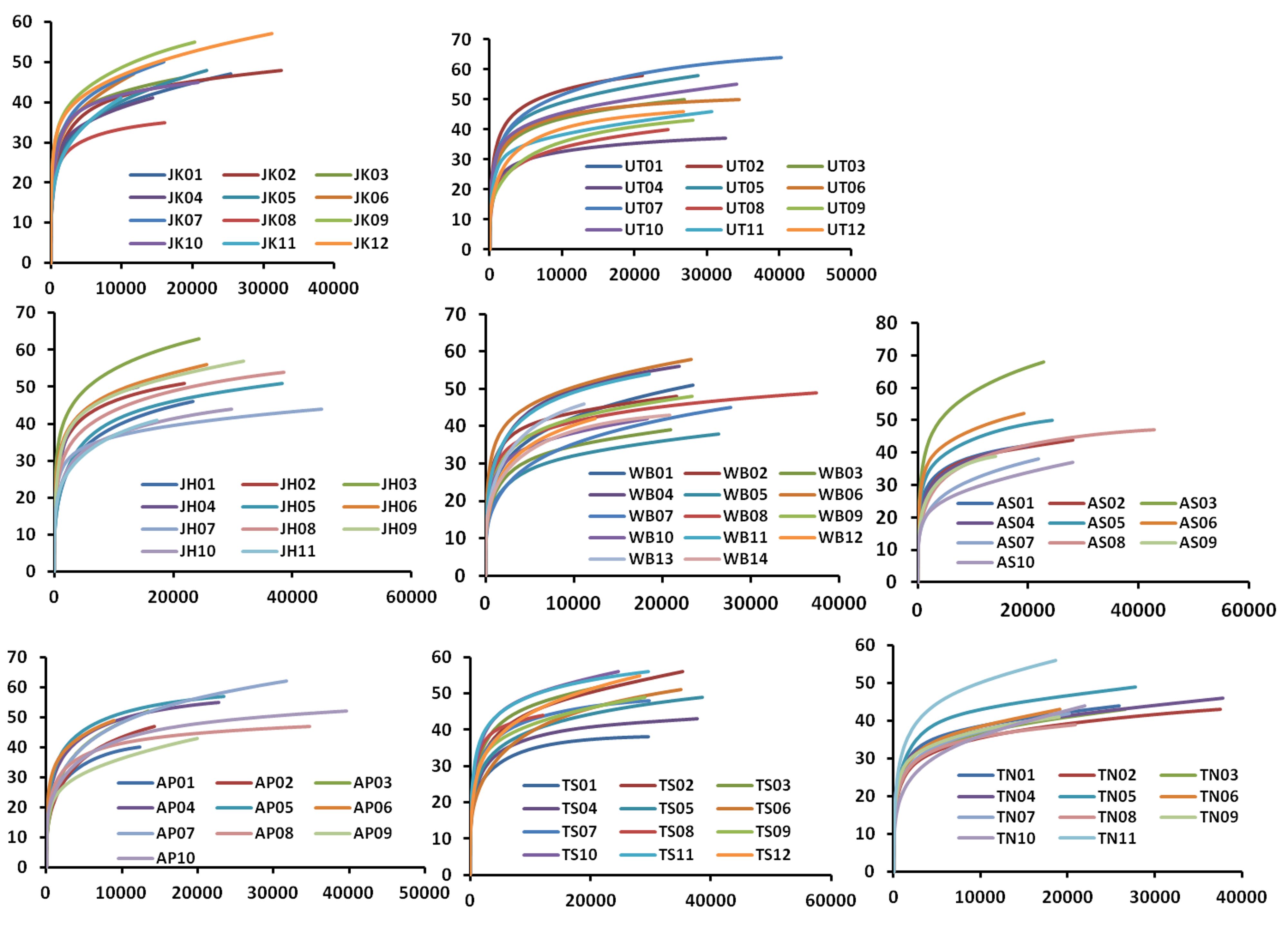

Supplement: S2 Fig — The analysis was carried out based on the abundance of various bacterial genera identified by sequencing the 16S rRNA gene (V1-V2 region) in each individual. The X-axis shows the number of randomly sampled sequences from each individual (one curve per individual) while Y-axis represents the mean bacterial richness based upon the bacterial genera identified. The sample code at the right bottom of each plot indicates the population. (TIF) [file pone.0184515.s002.tif]

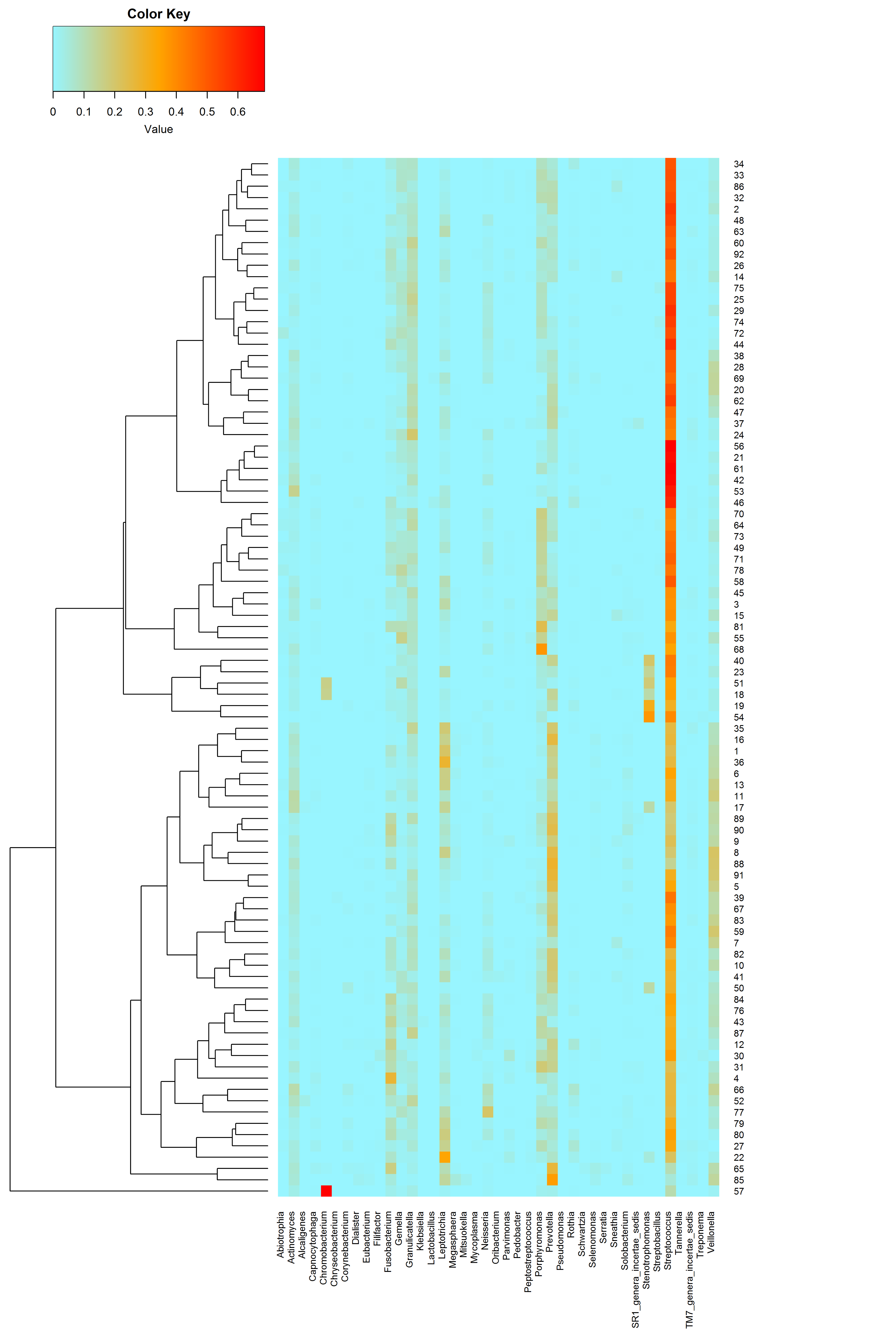

Supplement: S3 Fig — Bacterial genera with at least 1% abundance in a sample are represented in each column while individuals (N = 92) are clustered according to their relative distribution of bacterial genera. Colour key indicates the proportion of reads assigned to a genus for each sample. Sample codes; 1–12 (JK), 13–24 (UT), 25–35 (JH), 36–49 (WB), 50–59 (AS), 70–81 (TS) and 82–92 (TN). (TIF) [file pone.0184515.s003.tif]

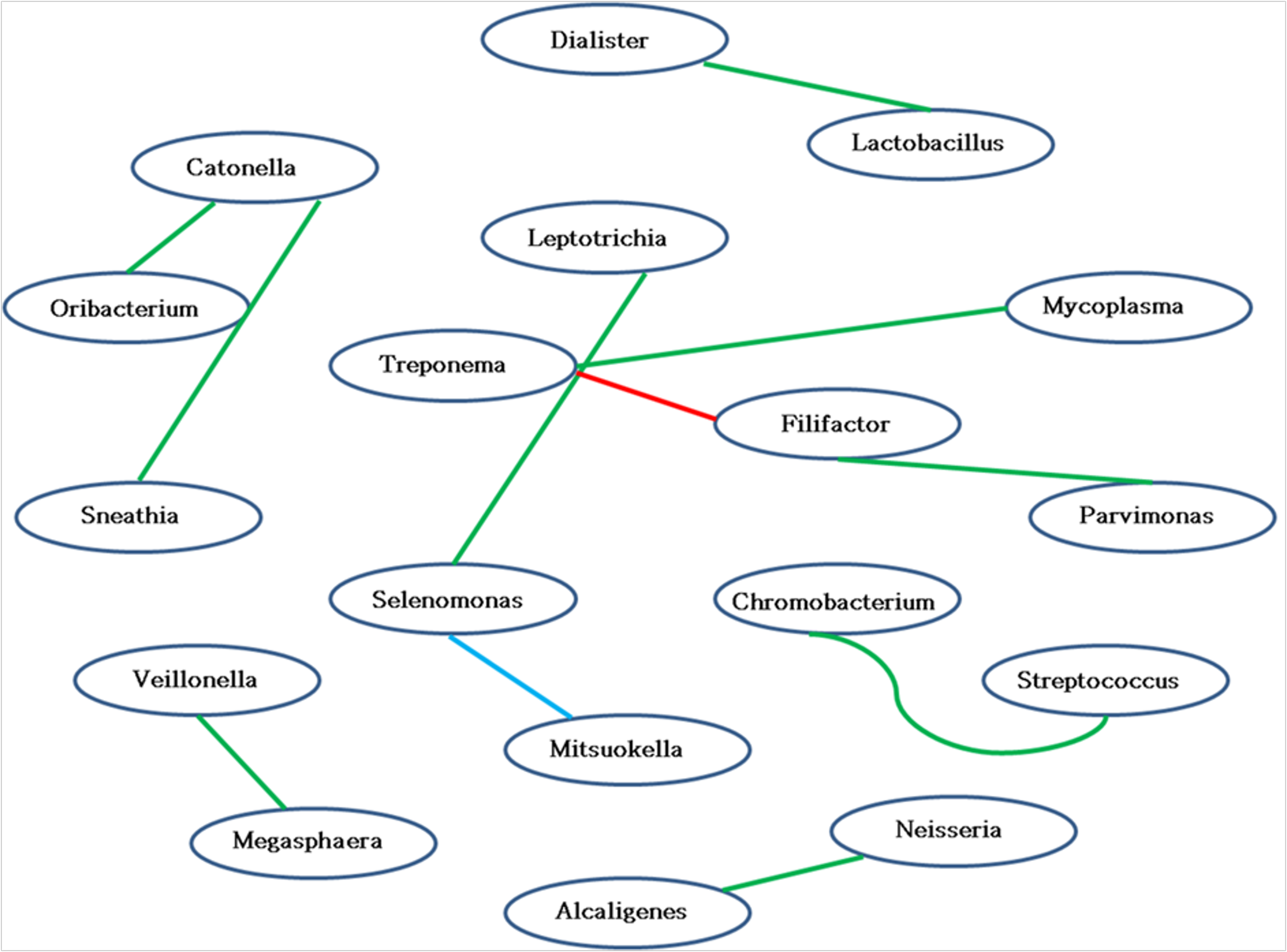

Supplement: S4 Fig — Straight line indicates positive interaction and curved line indicates negative interaction. Line colour denotes the corresponding regions: North (blue), East (green) and South (red). (TIF) [file pone.0184515.s004.tif]

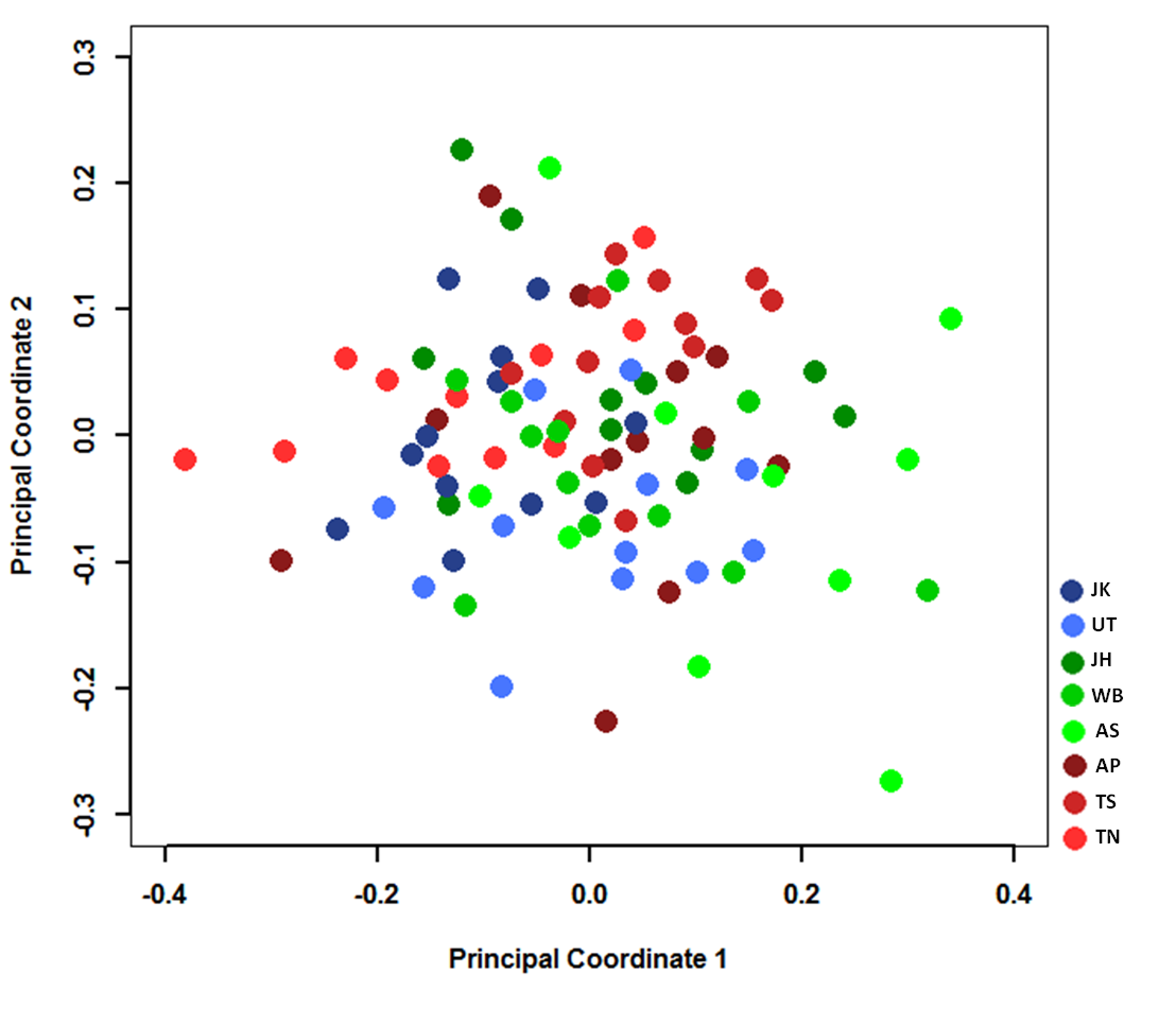

Supplement: S5 Fig — The first two components (PC1 and PC2) are shown here (Stress percentage = 17.5). Each sample is represented by a filled circle colored according to its geographical location. Populations from the same geographic region are represented by different shades of the same colour. (TIF) [file pone.0184515.s005.tif]

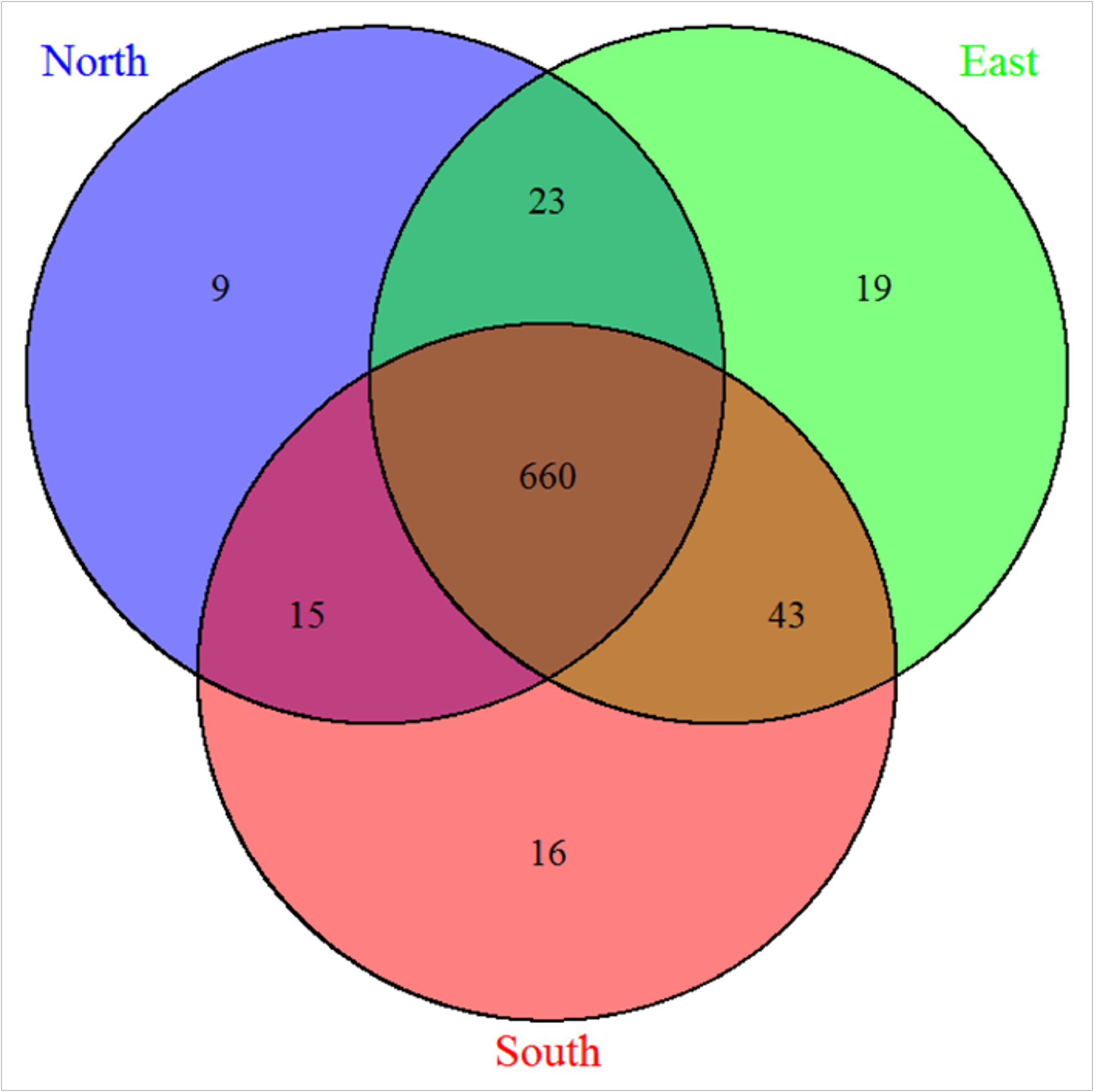

Supplement: S6 Fig — The distribution of 785 unique OTUs (obtained at 97% clustering) across North (blue), East (green) and South (red) India are shown. (TIF) [file pone.0184515.s006.tif]

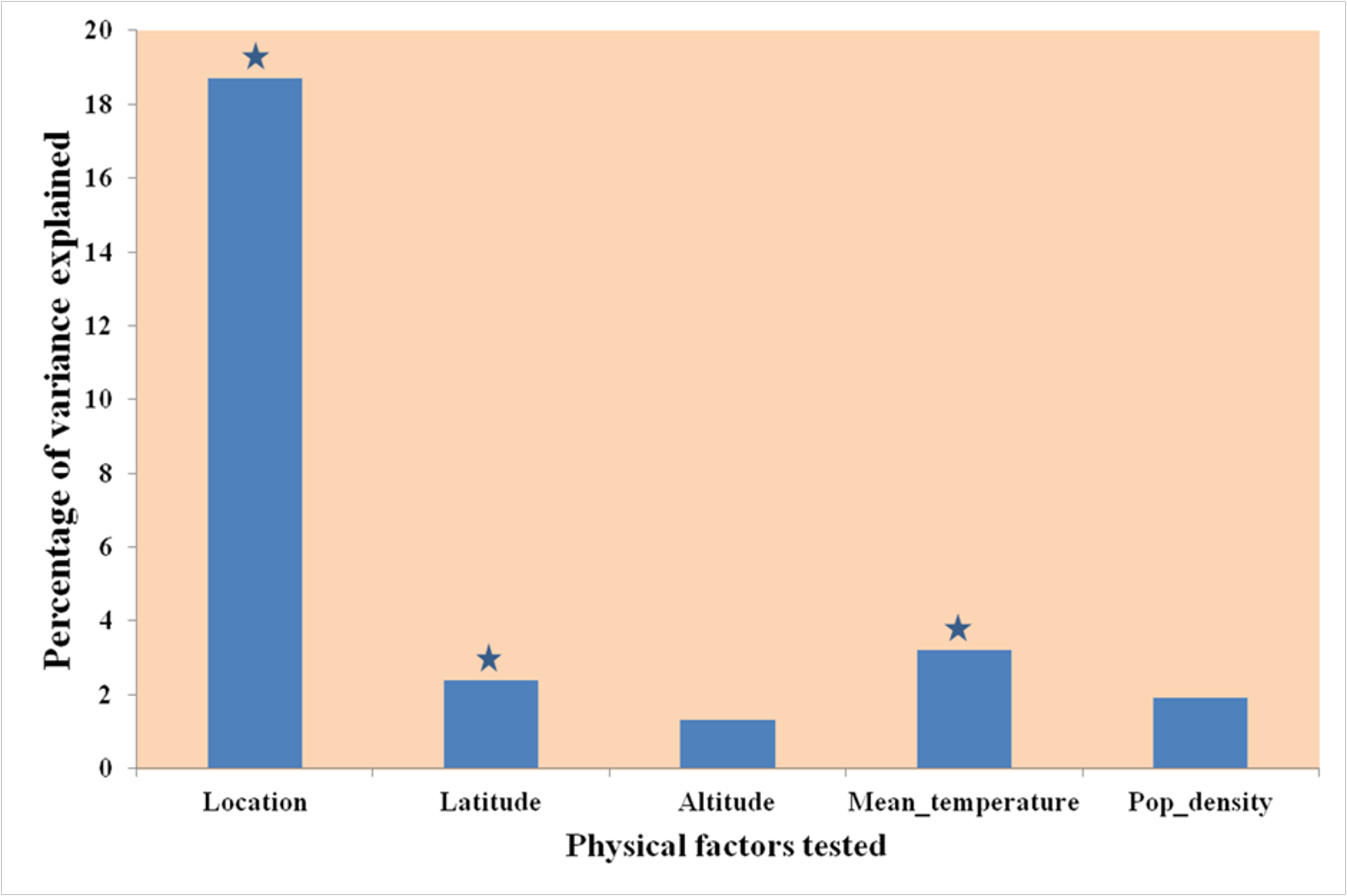

Supplement: S7 Fig — (*) shows the association was statistically significant (p<0.05). (TIF) [file pone.0184515.s007.tif]

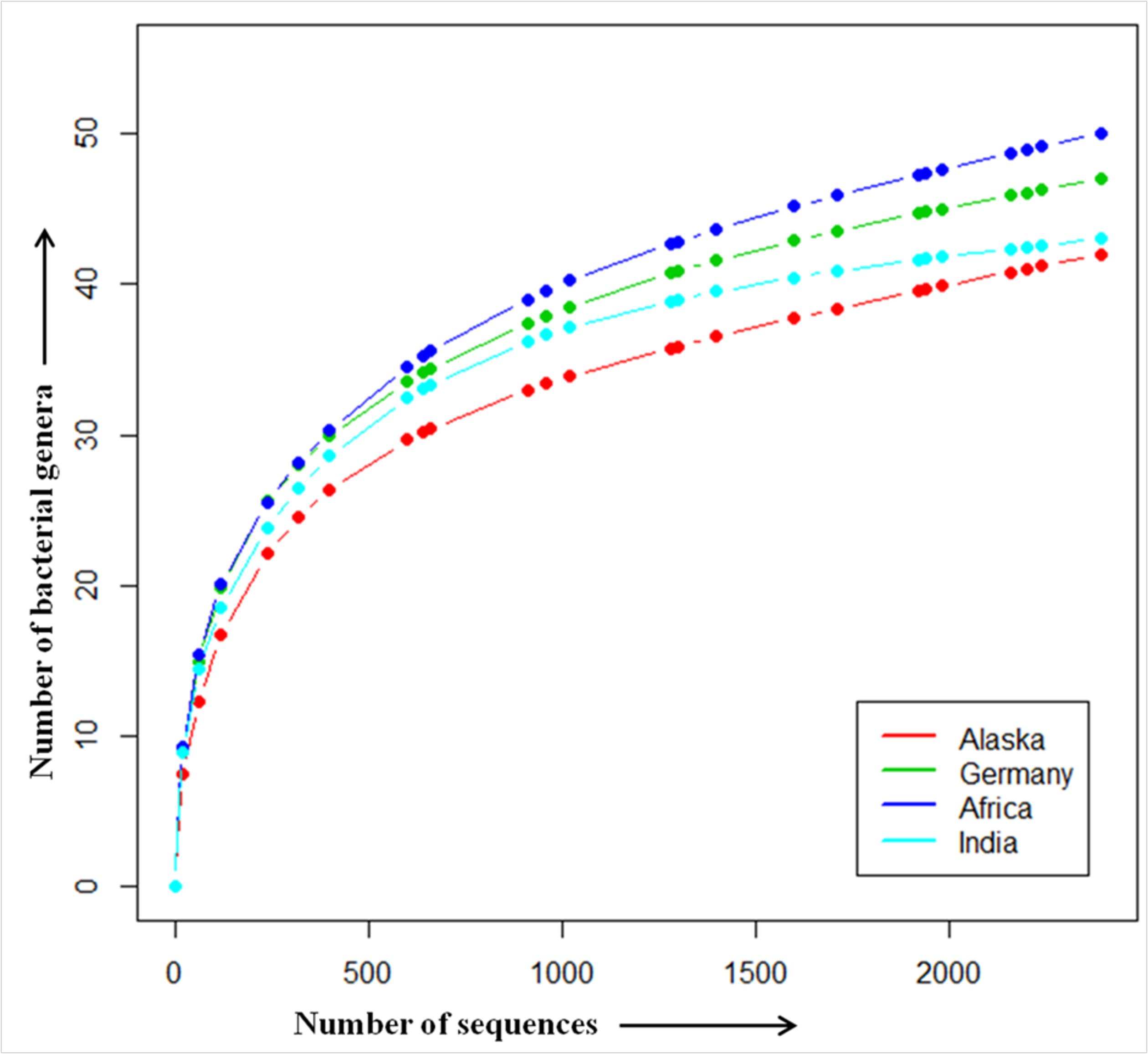

Supplement: S8 Fig — The analysis was carried out based on the abundance of various bacterial genera identified by sequencing of the partial 16S rRNA gene (V1-V2) in each individual. The X-axis shows the number of randomly sampled sequences from each population while Y-axis represents the mean bacterial richness based upon the bacterial genera identified. (TIF) [file pone.0184515.s008.tif]
