# Supplementary material for: Unraveling the human salivary microbiome diversity in Indian populations
Source: PLoS One. 2017 Sep 8;12(9):e0184515. doi: 10.1371/journal.pone.0184515 (PMC5590957; doi:10.1371/journal.pone.0184515)
Supplement: S1 Table — (DOCX) [file pone.0184515.s009.docx]

| **Sampling location** | **Location (State)** | **Code** | **Region** | **Latitude (in degrees)** | **Longitude**  **(in degrees)** | **Altitude (in metres)** | **Mean temperature (ºC)** | **Population density (per square kilometre)** |
| --- | --- | --- | --- | --- | --- | --- | --- | --- |
| Jammu | Jammu and Kashmir | JK | North | 33.45 | 76.57 | 327 | 24.2 | 124 |
| Garhwal | Uttarakhand | UT | North | 30.25 | 78.52 | 1650 | 27.5 | 129 |
| Dhanbad | Jharkhand | JH | East | 23.79 | 86.43 | 222 | 25.9 | 1284 |
| Kolkata | West Bengal | WB | East | 22.57 | 88.36 | 9 | 24.8 | 24252 |
| Guwahati | Assam | AS | East | 26.14 | 91.73 | 55.5 | 24.2 | 579 |
| Paderu | Andhra Pradesh | AP | South | 18.08 | 82.66 | 904 | 23.1 | 87 |
| Mahbubnagar | Telangana | TS | South | 16.46 | 78.11 | 498 | 35 | 219 |
| Chennai | Tamil Nadu | TN | South | 13.05 | 80.27 | 6 | 28.8 | 26553 |

**S1 Table: Details of the experimental variables at each sampling location used for geospatial analyses of salivary microbiome variation in India.**
