# Supplementary material for: Unraveling the human salivary microbiome diversity in Indian populations
Source: PLoS One. 2017 Sep 8;12(9):e0184515. doi: 10.1371/journal.pone.0184515 (PMC5590957; doi:10.1371/journal.pone.0184515)
Supplement: S2 Table — (DOCX) [file pone.0184515.s010.docx]

**S2 Table. Distribution of bacterial genera (> 0.05% in at least one location) and their corresponding tests of significance (p < 0.001, Sidak correction) for differential prevalence** (genera displaying significantly different prevalence are marked in bold).

| **Genera** | **JK** | **UT** | **JH** | **WB** | **AS** | **AP** | **TS** | **TN** | **p-value** |
| --- | --- | --- | --- | --- | --- | --- | --- | --- | --- |
| Abiotrophia | 0.138 | 0.308 | 0.169 | 0.314 | 0.290 | 0.459 | 0.882 | 0.238 | 0.057 |
| Actinomyces | 4.366 | 4.826 | 3.823 | 4.952 | 4.224 | 5.188 | 1.742 | 3.671 | 0.008 |
| Alcaligenes | 0.000 | 0.102 | 0.000 | 0.000 | 0.295 | 0.000 | 0.000 | 0.000 | 0.190 |
| **Atopobium** | 0.093 | 0.071 | 0.000 | 0.060 | 0.057 | 0.067 | 0.000 | 0.215 | **0.001** |
| Bacillus | 0.000 | 0.000 | 0.000 | 0.000 | 0.000 | 0.000 | 0.138 | 0.000 | 0.009 |
| Campylobacter | 0.061 | 0.000 | 0.000 | 0.065 | 0.000 | 0.000 | 0.000 | 0.098 | 0.085 |
| Capnocytophaga | 0.585 | 0.185 | 0.426 | 0.450 | 0.292 | 0.614 | 0.648 | 0.313 | 0.064 |
| Catonella | 0.164 | 0.114 | 0.115 | 0.158 | 0.000 | 0.093 | 0.080 | 0.097 | 0.081 |
| Chromobacterium | 0.000 | 1.542 | 0.000 | 0.000 | 13.835 | 0.000 | 0.000 | 0.000 | 0.016 |
| Chryseobacterium | 0.000 | 0.000 | 0.000 | 0.082 | 0.000 | 0.000 | 0.000 | 0.000 | 0.178 |
| Corynebacterium | 0.228 | 0.291 | 0.495 | 0.241 | 0.547 | 0.918 | 0.277 | 0.074 | 0.017 |
| Dialister | 0.169 | 0.064 | 0.085 | 0.106 | 0.000 | 0.154 | 0.000 | 0.323 | 0.370 |
| Enterobacter | 0.000 | 0.000 | 0.000 | 0.000 | 0.063 | 0.000 | 0.000 | 0.000 | 0.003 |
| Eubacterium | 0.361 | 0.156 | 0.254 | 0.194 | 0.185 | 0.168 | 0.087 | 0.480 | 0.208 |
| Filifactor | 0.172 | 0.150 | 0.363 | 0.057 | 0.000 | 0.065 | 0.115 | 0.059 | 0.065 |
| Fusobacterium | 7.133 | 2.473 | 4.421 | 4.717 | 1.488 | 3.534 | 5.825 | 7.050 | 0.001 |
| **Gemella** | 2.359 | 2.719 | 4.471 | 3.454 | 5.671 | 1.753 | 6.647 | 1.931 | **0.000** |
| Granulicatella | 5.462 | 6.751 | 7.713 | 6.782 | 6.340 | 8.153 | 7.947 | 6.088 | 0.503 |
| Klebsiella | 0.000 | 0.000 | 0.000 | 0.164 | 0.000 | 0.000 | 0.000 | 0.000 | 0.013 |
| Lactobacillus | 0.000 | 0.000 | 0.000 | 0.135 | 0.000 | 0.183 | 0.000 | 0.000 | 0.039 |
| Leptotrichia | 8.099 | 9.510 | 4.462 | 6.201 | 2.306 | 3.939 | 4.950 | 5.923 | 0.028 |
| **Megasphaera** | 0.620 | 0.300 | 0.082 | 0.131 | 0.127 | 0.146 | 0.079 | 1.118 | **0.001** |
| Mitsuokella | 0.000 | 0.102 | 0.000 | 0.000 | 0.000 | 0.000 | 0.000 | 0.284 | 0.004 |
| **Moryella** | 0.076 | 0.064 | 0.000 | 0.062 | 0.000 | 0.000 | 0.000 | 0.097 | **0.000** |
| Mycoplasma | 0.081 | 0.052 | 0.197 | 0.058 | 0.112 | 0.000 | 0.000 | 0.000 | 0.552 |
| **Neisseria** | 1.229 | 0.988 | 1.456 | 2.299 | 1.187 | 3.036 | 4.182 | 1.987 | **0.001** |
| **Oribacterium** | 0.203 | 0.124 | 0.111 | 0.263 | 0.081 | 0.052 | 0.000 | 0.118 | **0.000** |
| Paludibacter | 0.088 | 0.000 | 0.057 | 0.000 | 0.053 | 0.000 | 0.000 | 0.000 | 0.278 |
| Parvimonas | 0.680 | 0.576 | 0.902 | 0.334 | 0.256 | 0.165 | 0.421 | 0.216 | 0.164 |
| Pedobacter | 0.000 | 0.000 | 0.000 | 0.082 | 0.000 | 0.000 | 0.000 | 0.000 | 0.128 |
| Peptococcus | 0.087 | 0.119 | 0.178 | 0.082 | 0.122 | 0.000 | 0.085 | 0.075 | 0.300 |
| Peptostreptococcus | 0.401 | 0.356 | 0.495 | 0.286 | 0.346 | 0.306 | 0.145 | 0.373 | 0.555 |
| Porphyromonas | 4.282 | 2.804 | 9.916 | 5.110 | 5.012 | 8.003 | 11.316 | 5.365 | 0.001 |
| **Prevotella** | 15.053 | 11.308 | 8.224 | 8.762 | 5.282 | 8.772 | 5.052 | 19.937 | **0.000** |
| Pseudomonas | 0.000 | 0.000 | 0.000 | 0.064 | 0.000 | 0.000 | 0.000 | 0.000 | 0.717 |
| Rothia | 0.932 | 0.814 | 1.306 | 0.574 | 0.819 | 1.516 | 0.707 | 0.167 | 0.005 |
| Schwartzia | 0.000 | 0.000 | 0.052 | 0.000 | 0.000 | 0.104 | 0.000 | 0.000 | 0.099 |
| Selenomonas | 0.387 | 0.466 | 0.242 | 0.213 | 0.081 | 0.522 | 0.083 | 0.484 | 0.001 |
| Serratia | 0.000 | 0.000 | 0.000 | 0.056 | 0.000 | 0.070 | 0.000 | 0.000 | 0.195 |
| Sneathia | 0.436 | 0.607 | 0.326 | 0.141 | 0.000 | 0.000 | 0.220 | 0.391 | 0.020 |
| Solobacterium | 0.608 | 0.562 | 0.308 | 0.280 | 0.309 | 0.398 | 0.252 | 1.406 | 0.027 |
| SR1_genera_incertae_sedis | 0.100 | 0.089 | 0.154 | 0.286 | 0.138 | 0.077 | 0.272 | 0.199 | 0.197 |
| **Stenotrophomonas** | 0.000 | 7.520 | 0.057 | 1.759 | 7.048 | 0.000 | 0.000 | 0.000 | **0.001** |
| **Streptobacillus** | 0.000 | 0.000 | 0.000 | 0.000 | 0.069 | 0.090 | 0.641 | 0.000 | **0.000** |
| Streptococcus | 33.613 | 36.659 | 43.892 | 44.595 | 37.223 | 41.710 | 43.293 | 28.709 | 0.044 |
| Tannerella | 0.271 | 0.119 | 0.251 | 0.120 | 0.129 | 0.075 | 0.102 | 0.138 | 0.479 |
| TM7_genera_incertae_sedis | 0.595 | 0.577 | 0.473 | 0.453 | 0.358 | 0.511 | 0.338 | 0.843 | 0.339 |
| Treponema | 0.097 | 0.061 | 0.222 | 0.052 | 0.133 | 0.000 | 0.055 | 0.060 | 0.240 |
| **Veillonella** | 10.460 | 6.014 | 3.813 | 5.490 | 5.019 | 8.595 | 2.980 | 11.201 | **0.000** |
