# Supplementary material for: Unraveling the human salivary microbiome diversity in Indian populations
Source: PLoS One. 2017 Sep 8;12(9):e0184515. doi: 10.1371/journal.pone.0184515 (PMC5590957; doi:10.1371/journal.pone.0184515)
Supplement: S3 Table — (DOCX) [file pone.0184515.s011.docx]

**S3 Table: Distribution of bacterial genera (> 0.05% in at least one region) among the geographic regions and their corresponding tests of significance (p < 0.001, Sidak correction)** (genera displaying significantly different prevalence are marked in bold).

| **Genera** | **North** | **East** | **South** | **p-value North_vs_East** | **p-value North_vs_South** | **p-value East_vs_South** | **p-value all3_regions** |
| --- | --- | --- | --- | --- | --- | --- | --- |
| Abiotrophia | 0.238 | 0.255 | 0.562 | 0.562 | 0.185 | 0.045 | 0.116 |
| Actinomyces | 4.637 | 4.339 | 3.268 | 0.643 | 0.024 | 0.104 | 0.072 |
| Alcaligenes | 0.060 | 0.084 | 0.000 | 0.272 | 0.029 | 0.198 | 0.091 |
| Atopobium | 0.080 | 0.000 | 0.092 | 0.014 | 0.004 | 0.515 | 0.009 |
| Bacillus | 0.000 | 0.000 | 0.057 | 0.299 | 0.103 | 0.381 | 0.236 |
| Campylobacter | 0.000 | 0.000 | 0.058 | 0.440 | 0.936 | 0.425 | 0.652 |
| Capnocytophaga | 0.350 | 0.397 | 0.530 | 0.131 | 0.015 | 0.213 | 0.043 |
| Catonella | 0.135 | 0.107 | 0.089 | 0.211 | 0.438 | 0.415 | 0.395 |
| Chromobacterium | 0.910 | 3.945 | 0.000 | 0.329 | 0.268 | 0.033 | 0.098 |
| Corynebacterium | 0.265 | 0.419 | 0.378 | 0.254 | 0.698 | 0.401 | 0.482 |
| Dialister | 0.107 | 0.081 | 0.160 | 0.537 | 0.759 | 0.946 | 0.863 |
| Eubacterium | 0.240 | 0.213 | 0.236 | 0.301 | 0.572 | 0.585 | 0.574 |
| Filifactor | 0.159 | 0.161 | 0.084 | 0.235 | 0.534 | 0.573 | 0.499 |
| Fusobacterium | 4.390 | 3.691 | 5.625 | 0.468 | 0.099 | 0.007 | 0.024 |
| Gemella | 2.571 | 4.451 | 3.838 | 0.004 | 0.357 | 0.127 | 0.022 |
| Granulicatella | 6.221 | 6.991 | 7.397 | 0.135 | 0.068 | 0.594 | 0.155 |
| Klebsiella | 0.000 | 0.068 | 0.000 | 0.019 | 0.315 | 0.092 | 0.032 |
| Lactobacillus | 0.000 | 0.064 | 0.000 | 0.311 | 0.660 | 0.092 | 0.227 |
| Leptotrichia | 8.929 | 4.467 | 5.002 | 0.006 | 0.017 | 0.496 | 0.013 |
| **Megasphaera** | 0.432 | 0.112 | 0.434 | **0.000** | 0.081 | 0.099 | 0.002 |
| Mitsuokella | 0.070 | 0.000 | 0.095 | 0.004 | 0.242 | 0.060 | 0.015 |
| **Moryella** | 0.069 | 0.000 | 0.000 | **0.000** | 0.063 | 0.043 | 0.001 |
| Mycoplasma | 0.064 | 0.123 | 0.000 | 0.595 | 0.837 | 0.468 | 0.741 |
| **Neisseria** | 1.087 | 1.679 | 3.170 | 0.294 | **0.000** | 0.008 | **0.001** |
| **Oribacterium** | 0.157 | 0.156 | 0.068 | 0.076 | **0.001** | 0.089 | 0.003 |
| **Genera** | **North** | **East** | **South** | **p-value North_vs_East** | **p-value North_vs_South** | **p-value East_vs_South** | **p-value all3_regions** |
| Parvimonas | 0.619 | 0.516 | 0.288 | 0.829 | 0.357 | 0.436 | 0.604 |
| Peptococcus | 0.106 | 0.128 | 0.069 | 0.757 | 0.627 | 0.380 | 0.677 |
| Peptostreptococcus | 0.374 | 0.378 | 0.261 | 0.217 | 0.175 | 0.868 | 0.344 |
| Porphyromonas | 3.412 | 6.808 | 8.519 | 0.023 | 0.001 | 0.232 | 0.004 |
| Prevotella | 12.848 | 7.577 | 10.856 | 0.002 | 0.113 | 0.092 | 0.008 |
| Rothia | 0.863 | 0.907 | 0.743 | 0.206 | 0.497 | 0.092 | 0.183 |
| Selenomonas | 0.434 | 0.186 | 0.327 | 0.001 | 0.037 | 0.401 | 0.007 |
| Sneathia | 0.537 | 0.167 | 0.230 | 0.017 | 0.616 | 0.020 | 0.026 |
| Solobacterium | 0.581 | 0.298 | 0.664 | 0.007 | 0.349 | 0.149 | 0.034 |
| SR1_genera_incertae_sedis | 0.093 | 0.196 | 0.197 | 0.643 | 0.094 | 0.115 | 0.156 |
| **Stenotrophomonas** | 4.429 | 2.655 | 0.000 | 0.256 | 0.009 | **0.000** | **0.000** |
| **Streptobacillus** | 0.000 | 0.000 | 0.294 | 0.001 | 0.085 | **0.000** | **0.000** |
| Streptococcus | 35.406 | 42.242 | 38.144 | 0.008 | 0.265 | 0.127 | 0.030 |
| Tannerella | 0.181 | 0.170 | 0.107 | 0.853 | 0.238 | 0.232 | 0.377 |
| TM7_genera_incertae_sedis | 0.584 | 0.433 | 0.547 | 0.090 | 0.245 | 0.619 | 0.236 |
| Treponema | 0.076 | 0.136 | 0.054 | 0.914 | 0.910 | 0.893 | 0.988 |
| Veillonella | 7.843 | 4.754 | 7.115 | 0.002 | 0.121 | 0.078 | 0.006 |
