# Supplementary material for: Unraveling the human salivary microbiome diversity in Indian populations
Source: PLoS One. 2017 Sep 8;12(9):e0184515. doi: 10.1371/journal.pone.0184515 (PMC5590957; doi:10.1371/journal.pone.0184515)
Supplement: S4 Table — (DOCX) [file pone.0184515.s012.docx]

**S4 Table: Comparison of the 37 OTUs obtained in all the samples with the core microbiome described in previous studies**

| **S.no.** | **OTU** | **Genus** | **Li et.al., 2014** | **Huse, 2012** | **Li et.al., 2013** |
| --- | --- | --- | --- | --- | --- |
| 1 | OTU_27 | *Actinomyces* | Yes | Yes | Yes |
| 2 | OTU_31 |  |  |  |  |
| 3 | OTU_82 |  |  |  |  |
| 4 | OTU_40 | *Alloprevotella* | No | No | No |
| 5 | OTU_3 | *Fusobacterium* | Yes | Yes | Yes |
| 6 | OTU_4 |  |  |  |  |
| 7 | OTU_19 | *Gemella* | No | Yes | Yes |
| 8 | OTU_23 |  |  |  |  |
| 9 | OTU_24 |  |  |  |  |
| 10 | OTU_1 | *Granulicatella* | No | Yes | No |
| 11 | OTU_2 |  |  |  |  |
| 12 | OTU_109 | *Lachnoanaerobaculum* | No | No | No |
| 13 | OTU_36 | *Leptotrichia* | Yes | Yes | No |
| 14 | OTU_6 | *Porphyromonas* | Yes | No | Yes |
| 15 | OTU_8 |  |  |  |  |
| 16 | OTU_11 | *Prevotella* | Yes | Yes | Yes |
| 17 | OTU_287 |  |  |  |  |
| 18 | OTU_5 |  |  |  |  |
| 19 | OTU_50 | *Solobacterium* | No | No | No |
| 20 | OTU_108 | *Streptococcus* | Yes | Yes | Yes |
| 21 | OTU_114 |  |  |  |  |
| 22 | OTU_13 |  |  |  |  |
| 23 | OTU_14 |  |  |  |  |
| 24 | OTU_17 |  |  |  |  |
| 25 | OTU_216 |  |  |  |  |
| 26 | OTU_229 |  |  |  |  |
| 27 | OTU_28 |  |  |  |  |
| 28 | OTU_29 |  |  |  |  |
| 29 | OTU_41 |  |  |  |  |
| 30 | OTU_42 |  |  |  |  |
| 31 | OTU_53 |  |  |  |  |
| 32 | OTU_775 |  |  |  |  |
| 33 | OTU_780 |  |  |  |  |
| 34 | OTU_91 |  |  |  |  |
| 35 | OTU_97 |  |  |  |  |
| 36 | OTU_25 | *Veillonella* | Yes | Yes | No |
| 37 | OTU_26 |  |  |  |  |
